# Mind and the Emotions
*"A Commentary of Medical and Moral Life; or, Mind and the Emotions considered in relation to Health, Disease, and Religion." By William Cooke, M.D., M.R.C.S., editor of "Morgagni on the Seats and Causes of Disease," with Notes; author of "A Practical Treatise on Diseases of the Digestive Organs," &c. &c. London.


**Published:** 1853-01-01

**Authors:** 


### Art. IV.—MIND AND THE EMOTIONS.*

“ Mind and the Emotions,” is the attractive title emblazoned on the cover of this work. “Mind and the Emotions, by an editor of *Morgagni on the Seats and Causes of Disease*,” was an announcement calculated to arrest the attention of persons far less devoted than ourselves to psycho-

* “ A Commentary of Medical and Moral Life; or, Mind and the Emotions considered in relation to Health, Disease, and Religion.” By William Cooke, M.D., M.R.C.S., editor of “ Morgagni on the Seats and Causes of Disease,” with Notes; author of “ A Practical Treatise on Diseases of the Digestive Organs,” &c. &c. London.

logical pursuits. We referred to its pages with delight, but regret to add that we have met with disappointment. The author treads the beaten path of Millingen, Moore, and other recent popular writers on psychology—wanting, however, the originality of one and the learning of the other. Even the title of his book is a close paraphrase upon that of Dr. Millingen's "The Passions, or Mind and Matter illustrated by Considerations on Hereditary Insanity," and its contents in a great part carry out the paraphrase, except where the present author reveals to his readers the novel and astounding fact, that "religion takes possession of the emotions." In this phraseology, however, the author is original, for in passing the various passions in review (emotions, we should have written), he commences his paragraphs in the above manner, while his reasonings only show that the emotions are *influenced* by religion, and some of his illustrations partake of a character which we are reluctant to define. We will not select the most offensive of these, but the following will furnish the reader with an idea of the general tone of the work. The italics are our own. "Religion takes possession of the desire of the unhappiness of those we hate." . . . . . . . . "An *excellent* clergyman had heard reports concerning a Christian minister of another communion, and had probably read his publications; there was nothing in them derogatory to moral character, nor *any essential difference in doctrinal opinion*, and yet the *good* clergyman allowed a sentiment of strong dislike to arise within him. He remarked *to a pious parishioner*, 'I should like *to join the Evangelical* Alliance, but I shall meet Mr. ——, *whom I greatly hate.*' . . . . . . Some time afterwards the clergyman again met the same parishioner, and said, 'Well, I joined the alliance, and there I met Mr. ——. I had not been long in his company before I perceived *there was much* to admire in him, and soon afterwards I could not help loving him.' *Here* was the *true working* of Christian principle, in changing hatred into love; and it shows the advantage of communion among Christians who differ on minor points, and can conscientiously retain their differences without any impediment to their profitable intercourse," p. 199. Has not such a transformation of feeling as the above been often effected in social life, apart from "religion taking possession of the desire of the unhappiness of those we hate"? The feeling of desire is copiously illustrated in "evangelical" phraseology, from the lives of Adam, Habakkuk, Paul, Daniel, and David; of the latter of whom we are told "David, the sweet psalmist, appears sometimes as if he were actuated by the spirit of revenge. 'God,' says he, 'shall let me see my desire upon mine enemies.' And, as if that were not enough, he exultingly adds, 'Mine eye also shall see my *desire* upon mine enemies, and mine ears shall hear my *desire* of the wicked that rise up against me.'. We cannot wholly exempt even David from human infirmity, although we must admit that often when expressing himself as *desirous* that revenge may prey on the wicked, he is only uttering predictions of the evil that will come upon them," p. 200.

It is difficult to characterize the volume from which the above extracts are taken. It is neither a "commentary upon medical and moral life," in the sense which these words ordinarily convey, nor is it an original treatise on "mind and the emotions." After defining "medical life" and "moral life" in a manner *sui generis*, the author proceeds to give an autobiographical sketch of himself, as illustrating "the advantages and disadvantages" which he has had in reference to a proper elucidation of "these momentous subjects." Among the advantages for a due understanding of them, he *especially* enumerates "abiding in the country long enough to become familiar with things that have life—domestic animals, and the wild animals of the locality; the reptiles, and the birds; the fishes, and the insects; *also with fruits, vegetables, grains, grasses, and wild flowers, with underwood, and with trees*," p. iv. The "disadvantages" are only hinted at, they are not detailed. Judging from the result, we fear that the "disadvantages" greatly preponderated, and more than counterbalanced the "medical" and "moral" facts which the author derived from the "reptiles," and "wild flowers," and "underwood" of the country. As we have written above, we scarcely know how to characterize the work. Our reverence for the sublime truths of Christianity makes us slow to impugn the conduct of those who, blinded by zeal, use the language and recite the incidents of sacred writ with unconscious ostentation and irreverence. The hypocrite may be denounced; the zealot demands a wise and compassionate sympathy. Hence our embarrassment. This book consists of seven chapters—one of "Introductory Views," and six others of a purely elementary character on Anatomy and Psychology. The introductory views, extending over thirty-seven closely-printed pages, have special and exclusive reference *to medical men, and yet they are followed by a chapter on anatomy, which might have been written by any frequenter of a mechanics' institution.* This chapter, embracing a brief, loose, and general account of all the more important organs of the human body, is dignified by the title of "The Material Instruments of Mental and Moral Action," but no proof is given that they are the instruments of such extensive results. The five chapters which follow partake of a like elementary character, but, as we have shown, are laden with scripture phrases.

The introductory view "might form a very good penny pamphlet, to be distributed by the Religious Tract Society. It contains a few passing observations upon the value of phsychology to a medical man and the modification of religious feelings by temperament; but its chief aim is to show how the religious feeling may be brought to bear upon the medical treatment of invalids, and how necessary it is that medical men should themselves be religious. In the latter part, there is much indirect self-laudation, and some unnecessary condemnation of others. In the first part, the author, like many other amiable enthusiasts, seizes upon certain facts and arguments to uphold a dogma, which is wholly antagonistic to other dogmas of equal value in his esteem. Thus, at page 18, we are told of a poor fellow (who, while suffering from strangulated hernia, had positively refused to undergo an operation), that he was brought to consent to it by "arguments derivable from religious responsibility." He emphatically said, "I will die rather than submit!" The surgeons and pupils were leaving the ward as another surgeon, remarkable for his tact, entered; the case was mentioned to him, and the writer and a few other students accompanied him to the bedside of the patient. He spoke kindly to the man, who said: "They want me to have an operation performed, but I had rather die." "Well, well, my good fellow," said the surgeon, "I am very sorry it is necessary, but have you thought what there is after death? there is a judgment, and you must give an account of yourself to God. *God has been pleased to give us means to use, and it is our duty to use them; if you refuse to use the means God has given, and which we think* may save your life, you are in a measure answerable for your death, and must account to God for this sin with your other sins." At page 20—a lady suffering from cysto-sarcoma—"scornfully rejects *the use* of ether, previous to an operation, and wins high praise from our author by her conduct, and for having reached the "high attainment of enduring acute suffering of body with patience." Her case is brought forward to illustrate the truth, that "nothing conduces so greatly to promote such attainments as a sense of the supporting hand of God." These illustrations are always dangerously brought forward as special proofs of divine interposition and aid, for other persons, who are more intimately acquainted with the "Mind and the Emotions," than the author appears to be, could furnish from *the records of Paganism* stronger proofs of triumphant disdain of physical suffering, or to use a more orthodox phrase, "patience under suffering," than any of those which he has brought forward as illustrative of "the supporting hand of God." We need only briefly refer to the *ecstasy* under which the Hindoo widows could bear the torments of flame, and many of the devotees of Juggernaut, and other heathen deities, tolerate the most painful lacerations. The fearful mental epidemics, which have been so graphically described by Hecker, will also demonstrate certain conditions of the *emotions* under which individuals will bear an amount of physical infliction, to which the operations for the removal of a sarcomatous breast, so vauntingly introduced, are insignificant. We deny not the consolations of religion, but as psychologists we are bound to recognise in the disregard of physical suffering a certain condition of the nervous organism, *which can be induced* by the operation of *fixed laws,* apart from any *special* or immediate *miraculous* power; and which condition may be enforced as *potentially* by the *religious emotions* of a devout Hindoo or the pride of an Indian warrior as through the feelings which are praised. and justly praised, in the work before us. We are jealous, lest the incautious inferences of too zealous minds may furnish counter arguments for the scoffer. The "faith, which was once delivered to the saints" needs not the support of such inferences, and is wholly independent of such seeming miracles. We stated, that there was unfair condemnation of others. At page 33, we read—" A medical gentleman, who had devoted much time to the study of anatomy, particularly to what *is termed morbid anatomy,* remarked, 'I can perceive much to admire in nature, but cannot at all comprehend the subject of death. I cannot see why people should die.' " Had he studied the Epistles of Paul half as much as he had studied the writings of John Hunter, or ever read attentively 1 Cor. xv. he would have learned the design God had in the removal of mankind by death from time to eternity. This gentleman's case is worthy of remark. It shows the possibility of having the mind unceasingly occupied, through a long succession of years on subjects directly connected with death, without any serious self-application." This inference cannot fairly be deduced from the quoted expressions. The student "of what is termed morbid anatomy" may not have been able to *comprehend* the subject of death, philosophically. He may not have been able to detect the physical cause of death—or "*why*" people should die, and yet be free from the levity, indifference, and folly with which he is charged. Of who, or what he was, we know nothing, but the quoted expressions do not justify the condemnatory phrases. As the paragraph stands, it portrays a modest philosopher, who thought, as thousands have thought before him, and as tens of thousands will continue to think :

"How *wonderful* is Death!
Death and his brother Sleep—
One, pale as yonder waning moon
With lips of lurid blue;
The other, rosy as the morn,
When throned on Ocean's wave,
It blushes o'er the world:
*Yet both so passing wonderful.*"

This anecdote, and many others of a like character, together with frequent intimations of the want of religious conviction among medical men, and many direct and indirect references to the orthodox faith and personal piety of the author, prompted us to think that the volume before us was one among many popular treatises, having no higher aim than that of advertising the author, and we were prepared to denounce it accordingly. Further reflection, however, induces us to hope that, the religious sentiments and scriptural phrases which pervade the work, are the reflex of those which animate the heart and fill the mind of the writer, and although we cannot state that the book adds a single new truth to the science of psychology, yet we may conscientiously recommend its perusal to those who have hitherto never reflected upon the "mind and the emotions."

---

### Original Communications.

---

